# Yang Deficiency Body Constitution Acts as a Predictor of Diabetic Retinopathy in Patients with Type 2 Diabetes: Taichung Diabetic Body Constitution Study

**DOI:** 10.1155/2015/940898

**Published:** 2015-06-08

**Authors:** Cheng-Hung Lee, Tsai-Chung Li, Chia-I Tsai, Shih-Yi Lin, I-Te Lee, Hsin-Jung Lee, Ya-Chi Wu, Yi-Chang Su

**Affiliations:** ^1^Graduate Institute of Chinese Medicine, College of Chinese Medicine, China Medical University, Taichung, Taiwan; ^2^Department of Traditional Chinese Medicine, Han Ming Hospital, Changhua, Taiwan; ^3^School of Chinese Medicine, College of Chinese Medicine, China Medical University, Taichung, Taiwan; ^4^Graduate Institute of Biostatistics, China Medical University, Taichung, Taiwan; ^5^Department of Health Administration, College of Health Science, Asian University, Taichung, Taiwan; ^6^Department of Traditional Chinese Medicine, Taichung Veterans General Hospital, Taichung, Taiwan; ^7^Division of Endocrinology and Metabolism, Department of Internal Medicine, Taichung Veterans General Hospital, Taichung, Taiwan; ^8^Institute of Medicine, Chung Shan Medical University, Taichung, Taiwan; ^9^School of Medicine, National Yang-Ming University, Taipei, Taiwan; ^10^Division of New Drugs, Center for Drug Evaluation, Taipei, Taiwan

## Abstract

*Objective*. Diabetic retinopathy (DR), the most common microvascular complication of diabetes mellitus (DM), can cause severe visual impairment and blindness. To prevent the development of DR, identifying the associated risk factors for patient classification is critical. We conducted a cross-sectional study to determine whether body constitution (BC) is an independent predictor of DR. *Method*. 673 type 2 DM (T2DM) patients were recruited from a medical center, all received DR examination and body constitution questionnaire to assess BC. Other risk factors for DR were also recorded, including life style, history of diabetes, and blood pressure, etc. Multiple logistic regression analysis was conducted to calculate the odds ratios (ORs) for DR. *Results*. The prevalence of DR was significantly lower in Yang deficiency patients compared with non-Yang deficiency patients (24.69% versus 38.18% *P* = 0.02). After adjusting for other risk factors, we observed that patients exhibiting Yang deficiency BC were less likely to present with DR (OR = 0.531; 95% confidence interval = 0.312–0.903, *P* = 0.018). *Conclusion*. In addition to traditional risk factors, Yang deficiency BC might be an independent predictor of DR among T2DM patients and the results can be used as evidence for traditional Chinese medicine patient classification.

## 1. Introduction

Diabetic retinopathy (DR), the most common microvascular complication of diabetes mellitus (DM), has become a major cause of visual impairment and blindness worldwide [1–4], thus imposing a heavy burden on health care systems [5, 6]. The number of people with DM worldwide is estimated to continue increasing from 171 million in 2010 to 366 million in 2030 [7]; the prevalence of DR may also rise and cause an even greater socioeconomic burden [8]. The WHO multinational study on vascular disease in diabetes revealed that Chinese diabetes populations have a higher prevalence of DR [9]. Hence, reducing the risk of DR is an essential health concern for the ethnic Chinese diabetes populations in Taiwan, Hong Kong, Singapore, and mainland China, which comprise more than 25% of the global population.

The pathogenesis of DR is complex and not fully understood, and considerable efforts have been expended in identifying the possible risk factors for the development and progression of this disease [10–15]. Effective control of blood pressure and serum glucose and early detection and timely treatment of DR have been suggested to reduce the risk of DR-related vision loss [16–18]. However, new strategies are still necessary for further and substantial reduction of the DR risk [19, 20].

Traditional Chinese medicine (TCM), one of the most important and frequently used types of complementary and alternative medicine [21–23], is an ancient system of personalized medicine based on body constitution (BC) theory [24–26]. BC is the fundamental physiological component of a person, and different BC types are variously susceptible to disease and affect the development and prognosis of diseases [27, 28]. Patient classification is important in TCM, and different prevention and therapeutic methods for the same disease are used according to the BC type [29–31]; this is known as* tong bing yi zhi* in Chinese.

The relationships between BC and DM [32, 33], insulin resistance [34], diabetic nephropathy [35], and diabetic peripheral arterial disease [36] have been established, thus confirming TCM patient classification theory and verifying a new TCM treatment strategy for DM [37]. However, the association between BC and DR has yet to be determined. In the current study, we recruited type 2 DM (T2DM) patients and collected their BC types, a series of laboratory data, and fundus photographs for DR detection. We aimed to determine whether BC could be an independent predictor of DR in T2DM patients in ethnic Chinese populations.

## 2. Materials and Methods

### 2.1. Study Design and Subjects

**Figure 1:**
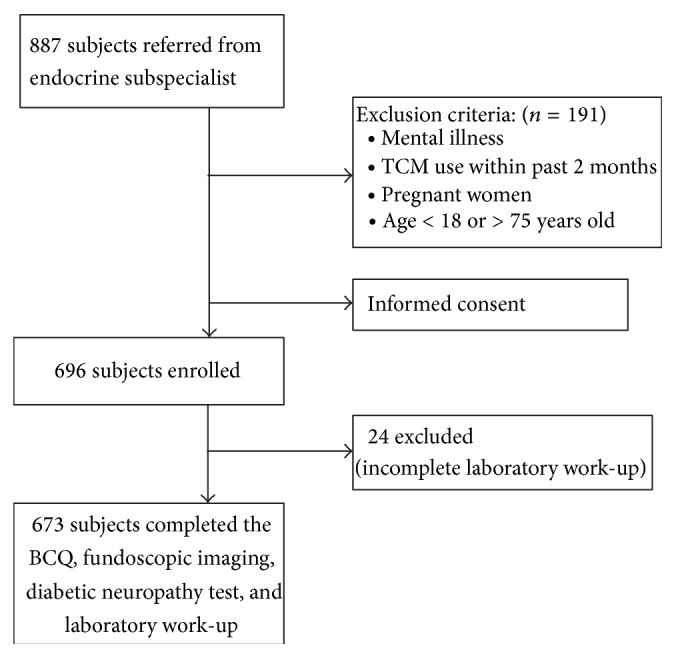
The flowchart of the study.

We conducted this cross-sectional study form February 2010 to February 2011 at the Diabetes Health Promotion Center of Taichung Veterans General Hospital (Taichung, Taiwan). The study protocol was approved by the Institutional Review Board of Taichung Veterans General Hospital (C10007). A total of 887 individuals diagnosed with T2DM were referred by endocrinology and metabolism subspecialists from an outpatient clinic, and 191 subjects older than 75 years were excluded. Written informed consent was obtained from each subject prior to participation in the study. Every subject received a diabetic retinopathy examination and was included in the enrollment group only when the possible risk factors for DR were completely collected: body constitution measurement, sociodemographic history (including sex, age, body mass index, and waist circumference), lifestyle, diabetic related history, blood pressure, lipid profile, renal parameters for diabetic nephropathy, and diabetic neuropathy examining results. Twenty-four subjects were excluded because of incomplete laboratory tests, and 673 T2DM patients were included in the final analysis.  Figure 1 shows the recruitment of the study subjects.

### 2.2. Measurements

#### 2.2.1. Body Constitution Measurement

All of the participants were administered a body constitution questionnaire (BCQ) consisting of three independent constitution subscales, including 19 items on Yang deficiency [24, 38], 19 items on Yin deficiency [39, 40], and 16 items on Phlegm stasis [27]. Because some items belonging to these three scales overlapped, the BCQ comprised 44 items on a 5-point Likert-type scale from 1 (*never happened*) to 5 (*always happens*). The final score of each constitution was calculated by summing the scores of all items on each subscale, with a higher score implying a greater deviation from the constitution. The diagnostic cut-off points for Yang deficiency, Yin deficiency, and Phlegm stasis were 30.5 [38], 29.5 [40], and 26.5 [27], respectively. BCQ demonstrates favorable factorial validity [27], and Cronbach's *α* of each constitution subscale in previous studies has been between 0.88 and 0.90 [27, 38, 40].

#### 2.2.2. Detection of DR

Central fundus photographic imaging was performed following the standardized protocol. Both eyes of each subject were photographed using a nonstereoscopic 45° digital nonmydriatic camera (CR-DGi, Canon, Inc., Tokyo, Japan). The fundus photographs were examined in a masked manner by experienced and trained endocrinology and metabolism subspecialists. The DR severity of each eye was graded according to the International Clinical Diabetic Retinopathy and Diabetic Macular Edema Disease Severity Scales [41]. Because nonproliferative diabetic retinopathy (NPDR) and proliferative diabetic retinopathy (PDR) were considered early and late stages of DR, respectively, study participants who had at least one eye with either NPDR or PDR were assigned to the DR group for analysis.

### 2.3. Data Collection

Several critical risk factors for DR were derived to control for the confounding influence. The sociodemographic and biological characteristics of the 673 participants, including sex, age, height, waist, lifestyle, duration of DM, oral hypoglycemic agent use, and insulin usage, were investigated through personal interviews at the Diabetes Health Promotion Center of Taichung Veterans General Hospital. After more than 12 hours of fasting, a series blood samples were collected for measuring fasting blood sugar, glycosylated hemoglobin (HbA1c), total cholesterol, total triglyceride, high density lipoprotein, low density lipoprotein (LDL), and creatinine (Cr). The estimated glomerular filtration rate (eGFR) was calculated using the Modification of Diet in Renal Disease four-variable equation: eGFR = 186 × serum creatinine − 1.154 × age − 0.203 × 1.212 (if black) × 0.742 (if female) [42]. We also collected a spot urine from each participant to analyze urine protein, and the estimated daily urine protein output was calculated using the following equation: albumin in spot urine/serum creatinine (ALB/Cr). A diabetic neuropathy examination was performed based on the physical examination protocol of the Michigan Neuropathy Screening Instrument (MNSI) [43].

### 2.4. Statistical Analysis

The data were presented as mean ± SD for continuous variables and as number (%) for categorical variables. Differences between groups were compared using a chi-square test for categorical variables and a* t*-test for continuous variables. We used multiple logistic regression analysis to calculate the odds ratios (ORs) for DR. Hierarchical models for covariant variables were considered for determining whether BC is an independent predictor of DR. First, crude ORs were calculated without adjustment. We then sequentially entered sociodemographic factors, lifestyle, diabetic factors, blood pressure, and lipid profiles into the model. Finally, renal parameters and diabetic neuropathy were added into the final model. A two-sided significance level was set at *P* < 0.05. We performed all analyses using SAS version (SAS Institute Inc., Cary, NC, USA).

## 3. Results 

A total of 343 (51%) males and 330 (49%) females composed the study group. Of the 673 participants, 81 (12%), 174 (25.9%), and 86 (12.8%) patients were diagnosed with Yang deficiency, Yin deficiency, and Phlegm stasis BC, respectively.  Table 1 shows a comparison of sociodemographic factors, lifestyle, diabetic factors, lipid profile, blood pressure, renal parameters, and diabetic neuropathy between subjects with and without Yang deficiency, Yin deficiency, and Phlegm stasis BC. Sex differs in all BCs. Patients with Phlegm stasis BC had higher BMI, LDL level, and lack of exercise habits as compared with the non-Phlegm stasis patients. Participants with Yang or Yin deficiency BC tended to have higher percentage of insulin usage. Among individuals with Yin deficiency BC, higher percentage of diabetic neuropathy and lower GFR level were also noted.

A total of 226 (33.6%) patients have DR, including PDR and NPDR.  Table 2 shows the prevalence of DR according to BC status. The prevalence of DR was significantly lower in Yang deficiency patients compared with non-Yang deficiency ones (24.69% versus 38.18%, *P* = 0.02).

The unadjusted and hierarchically adjusted ORs for DR associated with different BC were shown in Table 3. Individual with Yang deficiency BC was less likely to have DR (crude OR = 0.531; 95% CI = 0.312–0.903, *P* = 0.018). After adjusting for all of the variables, including Yin deficiency, Phlegm stasis, sociodemographic factors, lifestyle, diabetic factors, blood pressure, lipid profile, renal parameters, and diabetic neuropathy, Yang deficiency BC remained significantly associated with DR (OR = 0.453; 95% CI = 0.234–0.875, *P* = 0.019).

## 4. Discussion

Substantial efforts have been expended to discover the risk factors associated with DR among T2DM patients. Male sex, blood pressure, duration of diabetes, HbA1c, and albuminuria had been identified to be associated with DR among T2DM patients across different ethnic groups [11–13, 44, 45]. In our study, we considered all of the aforementioned risk factors as well as other confounding factors. After adjusting for other variables, this cross-sectional study suggests that Yang deficiency BC is an independent predictor of DR. T2DM patients with Yang deficiency BC had a 55% reduced likelihood of DR.

An individual's BC is formed by Yin and Yang and the imbalance between Yin and Yang renders individuals more prone to certain diseases [24, 46]. Yang consists of the energy for maintaining body function, and the diminishing energy level is defined as Yang deficiency. Several research and clinical trials have indicated the association between disease or discomfort and Yang deficiency [32, 47–51]. Persisting lack of Yin is called Ying deficiency[39], and Phlegm stasis means when the transportation of Yin and Yang is obstructed [52]. In our study, the results revealed that patients with Yang deficiency BC might have a lower risk of DR, and further research is necessary for exploring the mechanism of how Yang deficiency BC influences DR development in T2DM patients.

Hemodynamic abnormalities, including increased retinal blood flow and blood pressure, might lead to the development and progression of DR [53–57]. Yang deficiency is regarded as a decrease in energy level, including the force of circulation [38]. Traditional therapeutic methods of TCM for Yang deficiency are termed* yi qi wen yang* and demonstrate the effect of promoting blood circulation [58]. Blood pressure did not differ significantly between patients with or without Yang deficiency BC in this study and was considered in our multiple logistic regression analysis. Thus, the retinal blood flow of patients may explain the effect of Yang deficiency on DR observed in our study.

Angiogenesis is another vital pathogenic pathway in diabetic retinopathy [55, 57] and the effect of antivascular endothelial growth factor therapy has been clinically studied. Several studies have found that single-herb or herbal remedies used based on the principle of replenishing Qi and Yang, thus increasing the energy level, has the therapeutic effect of ischemic damage [59, 60] and wound healing [61] through angiogenesis. Whether patients with Yang deficiency BC have less angiogenesis or fewer vascular endothelial growth factors that might result in the decreased risk of DR is a topic of scientific interest.

Previous studies have successfully provided evidence for TCM patient classification by using machine learning algorithms [62–64], and by using epidemiology module, our study results confirm that people with certain BCs might be more susceptible to some diseases. We launched the Taichung Diabetic Body Constitution Study to evaluate the effect of BC on T2DM patients. Previous results have shown that T2DM patients with Yang deficiency, Yin deficiency, or Phlegm stasis BC had reduced health-related quality of life [65]. The BC concept can guide TCM physicians to treat patients according to different BCs and improve health conditions by helping patients to adjust their BC status. A previous randomized controlled trial reported that higher life quality, reduction of antihypertensive medication, and a significant difference in systolic blood pressure could be attained by using Chinese food therapy to restore Ying-Yang imbalance in hypertensive patients with Yin deficiency BC [66]. Studies have identified certain Chinese herbs that demonstrate antidiabetic [67–69] and antihyperglycemic [70, 71] effects, which can be useful in future clinical research when combined with TCM patient classification.

The questionnaire, BCQ, with favorable reliability and validity can be easily applied by Western physicians to assess the BC status and has been used in clinical research [36, 50]. In this study, we focused on the independent influence of BC on DR; hence, we considered other focal complications of DM, including diabetic neuropathy and nephropathy. Further clinical research based on the study results is necessary to investigate the effect of preventing or treating DR by adjusting BC.

Our study has three major limitations. First, the abnormal constitution theoretically occurs before a disease, but the time sequence of a cross-sectional study design cannot determine the causal relationship between BC and DR. A cohort study might be necessary to mitigate this doubt. Second, because the patients willing to join the study might have been more aggressive in improving their health conditions, a potential selection bias exists. To mitigate the impact of bias, we considered the lifestyle and sociodemographic factors in the multiple logistic regression analysis. Finally, although we included major confounding factors, other unmeasured variables might exist because this was an observational study.

## 5. Conclusion

Distinguishing T2DM patients who exhibit a high or low risk of complication development is crucial for health management. This study suggests that BC is independently associated with DR. T2DM patients with Yang deficiency BC had a 55% significantly reduced likelihood of DR. For patient classification, using BCQ to assess the BC is convenient, inexpensive, and noninvasive and should be adopted in clinical practice. Identifying the association between DR and BC confirms BCQ construct validity. Our study results might guide future study to survey the longitudinal association between DR and BC.

## Figures and Tables

**Table 1:** Participant characteristics.

	Yang deficiency (*n* = 673)			Yin deficiency (*n* = 673)			Phlegm stasis (*n* = 673)		
	Yes (*n* = 81)	No (*n* = 592)	*P* value	Yes (*n* = 174)	No (*n* = 499)	*P* value	Yes (*n* = 86)	No (*n* = 587)	*P* value
Age (years)	57.57 ± 11.97	59.21 ± 10.60	0.20	60.01 ± 11.18	58.67 ± 10.63	0.16	59.21 ± 11.13	58.98 ± 10.74	0.86
Female, *n* (%)	59 (72.84)	271 (45.78)	<0.001^‡^	100 (57.47)	230 (46.09)	<0.01^†^	57 (66.28)	273 (46.51)	<0.001^‡^
BMI (kg/m^2^)	26.05 ± 4.26	25.67 ± 4.07	0.44	25.64 ± 3.69	25.74 ± 4.23	0.76	27.11 ± 4.68	25.51 ± 3.97	<0.01^†^
WAIST (cm)	88.04 ± 10.77	89.01 ± 10.46	0.43	87.83 ± 10.10	89.27 ± 10.61	0.12	91.98 ± 11.93	88.44 ± 10.20	0.01^∗^
Lifestyle factors									
Smoke history, yes, *n* (%)	4 (4.93)	34 (5.74)	0.77	8 (4.59)	30 (6.01)	0.49	6 (6.97)	32 (5.45)	0.57
Alcohol usage, yes, *n* (%)	0 (0)	21 (3.54)	0.09	2 (1.14)	19 (3.80)	0.08	0 (0)	21 (3.57)	0.07
Exercise habits, yes, *n* (%)	59 (72.83)	475 (80.23)	0.12	132 (75.86)	402 (80.56)	0.19	56 (65.11)	478 (81.43)	<0.001^‡^
Diabetic factors									
FBS (mg/dL)	149.68 ± 60.61	144.98 ± 45.72	0.50	147.71 ± 53.87	144.8 ± 45.43	0.52	147.84 ± 41.99	145.21 ± 48.53	0.63
HbAlc (%)	7.87 ± 1.78	7.78 ± 1.67	0.67	7.86 ± 1.86	7.77 ± 1.61	0.58	8.08 ± 1.73	7.75 ± 1.67	0.08
DMH (year)	8.20 ± 7.40	8.28 ± 7.28	0.93	8.61 ± 7.34	8.15 ± 7.27	0.48	8.02 ± 6.82	8.31 ± 7.36	0.74
OHA use, yes, *n* (%)	75 (92.59)	562 (94.93)	0.38	165 (94.82)	472 (94.58)	0.90	81 (94.18)	556 (94.71)	0.84
Insulin usage, yes, *n* (%)	32 (39.51)	147 (24.83)	<0.01^†^	61 (35.06)	118 (23.65)	<0.01^†^	28 (32.56)	151 (25.72)	0.18
Lipid profile									
TC (mg/dL)	177.79 ± 39.74	175.46 ± 36.26	0.59	173.32 ± 35.84	176.58 ± 36.95	0.31	180.95 ± 41.93	174.97 ± 35.81	0.21
TG (mg/dL)	160.1 ± 120.29	156.82 ± 180.37	0.83	149.73 ± 91.91	159.82 ± 194.46	0.37	163.88 ± 136.23	156.24 ± 179.13	0.64
HDL (mg/dL)	52.24 ± 13.49	52.96 ± 14.78	0.68	51.68 ± 13.68	53.29 ± 14.93	0.21	51.51 ± 14.17	53.07 ± 14.69	0.36
LDL (mg/dL)	110.37 ± 31.97	106.9 ± 32.47	0.37	106.64 ± 33.85	107.56 ± 31.91	0.75	114.08 ± 36.41	106.33 ± 31.69	0.04^∗^
Blood pressure									
SBP (mmHg)	131.14 ± 17.05	131.58 ± 14.37	0.82	131.35 ± 15.57	131.59 ± 14.41	0.85	131.90 ± 16.21	131.47 ± 14.49	0.80
DBP (mmHg)	78.91 ± 11.31	78.38 ± 8.74	0.68	77.68 ± 9.93	78.70 ± 8.76	0.23	77.50 ± 10.09	78.58 ± 8.93	0.30
GPT (U/L)	29.28 ± 30.00	29.00 ± 23.53	0.94	28.43 ± 28.99	29.24 ± 22.57	0.74	32.52 ± 30.82	28.52 ± 23.27	0.25
Renal parameters									
Microalbumin (mg/dL)	27.85 ± 86.83	21.97 ± 72.16	0.56	28.12 ± 92.32	20.77 ± 66.49	0.34	27.47 ± 89.66	21.97 ± 71.52	0.59
Cr (mg/dL)	1.09 ± 0.46	1.14 ± 0.63	0.39	1.19 ± 0.63	1.12 ± 0.61	0.19	1.09 ± 0.49	1.14 ± 0.63	0.34
eGFR (ml/min)	69.56 ± 24.42	71.01 ± 21.52	0.57	67.22 ± 23.72	72.10 ± 21.07	0.01^∗^	70.64 ± 23.35	70.86 ± 21.67	0.93
ALB/CR (ug/mg)	475.24 ± 2008.6	233.91 ± 935.92	0.29	319.95 ± 1386.8	243.08 ± 1013.1	0.50	316.61 ± 1238.90	255.10 ± 1103.70	0.64
Diabetic neuropathy, yes, *n* (%)	63 (77.78)	14 (2.36)	0.08	48 (27.59)	29 (5.81)	0.01^∗^	64 (74.42)	13 (2.21)	0.25

Data were presented as mean ± SD for continuous variable and as number (%) for categorical variable.

*P* values were calculated using the chi-square test for categorical variable and *t*-test for continuous variable ^∗^ *P* < 0.05, ^†^ *P* < 0.01, and ^‡^ *P* < 0.001.

BMI, body mass index; FBS, fasting blood sugar; HbA1c, glycosylated hemoglobin; DMH, duration of diabetic mellitus; OHA, oral hypoglycemic agent; TC, total cholesterol; TG, total triacylglycerol; HDL, high density lipoprotein; LDL, low density lipoprotein; SBP, systolic blood pressure; DBP, diastolic blood pressure; GPT, glutamic pyruvic transaminase; Cr, creatinine; eGFR, estimated glomerular filtration rate; ALB/CR, microalbumin to creatinine ratio.

**Table 2:** Prevalence of diabetic retinopathy according to body constitution.

BC	DR	non-DR	Total	*P* value
	*n* (%)	*n* (%)	*N* (%)	
Yang deficiency				
Yes	20 (24.69)	61 (75.31)	81 (100)	0.02^∗^
No	226 (38.18)	366 (61.82)	592 (100)	
Yin deficiency				
Yes	62 (35.63)	112 (64.37)	174 (100)	0.77
No	184 (36.87)	315 (63.13)	499 (100)	
Phlegm stasis				
Yes	24 (27.91)	62 (72.09)	86 (100)	0.07
No	222 (37.82)	365 (62.18)	587 (100)	

BC: body constitution; DR: diabetic retinopathy. ^∗^ *P* < 0.05.

*P* values were calculated using the two-sided chi-square test.

**Table 3:** Unadjusted and adjusted odds ratios and 95% CI for diabetic retinopathy according to constitution.

	Diabetic retinopathy, OR (95% CI)					
	Yang deficiency		Ying deficiency		Phlegm stasis	
	OR (95% CI)	*P* value	OR (95% CI)	*P* value	OR (95% CI)	*P* value
Model 1	0.531 (0.312–0.903)	0.018^∗^	0.948 (0.662–1.358)	0.770	0.637 (0.386–1.049)	0.075
Model 2	0.536 (0.289–0.995)	0.048^∗^	1.263 (0.832–1.917)	0.273	0.735 (0.409–1.323)	0.305
Model 3	0.529 (0.282–0.995)	0.048^∗^	1.164 (0.761–1.780)	0.485	0.779 (0.427–1.422)	0.416
Model 4	0.531 (0.282–0.999)	0.049^∗^	1.154 (0.754–1.768)	0.510	0.746 (0.407–1.366)	0.342
Model 5	0.498 (0.262–0.947)	0.034^∗^	1.099 (0.712–1.697)	0.670	0.768 (0.415–1.421)	0.401
Model 6	0.472 (0.247–0.902)	0.023^∗^	1.108 (0.715–1.717)	0.645	0.812 (0.436–1.512)	0.511
Model 7	0.453 (0.234–0.875)	0.019^∗^	1.057 (0.677–1.648)	0.810	0.824 (0.438–1.550)	0.548

Model 1 is unadjusted. Model 2 is adjusted for BC. Model 3 is additionally adjusted for sociodemographic factors. Model 4 is additionally adjusted for lifestyle. Model 5 is additionally adjusted for diabetic factors. Model 6 is additionally adjusted for blood pressure and lipid profile. Model 7 is additionally adjusted for renal parameters and diabetic neuropathy.

Analysis by logistic regression. ^∗^ *P* < 0.05.

BC: body constitution, including Yang deficiency, Ying deficiency, and Phlegm stasis. Sociodemographic factors: gender, age, BMI, and waist. Lifestyle: smoke and alcohol drinking history and exercise. Diabetic factors: FBS, HbA1c, DM duration, oral hypoglycemia agent, and insulin use. Blood pressure: SBP and DBP. Lipid profile: TG, HDL, and LDL. Renal parameters: GFR and microalbumin to creatinine ratio.
